# Preparation of Specific Nanobodies and Their Application in the Rapid Detection of Nodularin-R in Water Samples

**DOI:** 10.3390/foods10112758

**Published:** 2021-11-10

**Authors:** Jinyi Yang, Rui Si, Guangpei Wu, Yu Wang, Ruyu Fang, Fei Liu, Feng Wang, Hongtao Lei, Yudong Shen, Qi Zhang, Hong Wang

**Affiliations:** 1 Guangdong Provincial Key Laboratory of Food Quality and Safety, National-Local Joint Engineering Research Center for Processing and Safety Control of Livestock and Poultry Products, College of Food Science, South China Agricultural University, Guangzhou 510642, China; yjy361@163.com (J.Y.); huanongxiaosi@163.com (R.S.); wgphavoc@163.com (G.W.); 18819259799@163.com (R.F.); m13476079515@163.com (F.L.); wangfeng_sp@163.com (F.W.); hongtao@scau.edu.cn (H.L.); shenyudong@scau.edu.cn (Y.S.); 2 Guangzhou Institute of Food Inspection, Guangzhou 510080, China; xxwangyu@163.com; 3 Oil Crops Research Institute, Chinese Academy of Agricultural Sciences, Xudong 2nd Road No. 2, Wuchang District, Wuhan 430062, China; zhangqi01@caas.cn

**Keywords:** nodularin, nanobody, detection, ic-ELISA

## Abstract

Nanobodies have several advantages, including great stability, sensibility, and ease of production; therefore, they have become important tools in immunoassays for chemical contaminants. In this manuscript, nanobodies for the detection of the toxin Nodularin-r (NOD-R), a secondary metabolite of cyanobacteria that could cause a safety risk for drinks and food for its strong hepatotoxicity, were for the first time selected from an immunized Bactrian camel VHH phage display library. Then, a sensitive indirect competitive enzyme-linked immunosorbent assay (ic-ELISA) for NOD-R, based on the nanobody N56 with great thermostability and organic solvent tolerance, was established under optimized conditions. The results showed that the limit of detection for NOD-R was 0.67 µg/L, and the average spike recovery rate was between 84.0 and 118.3%. Moreover, the ic-ELISA method was validated with spiked water sample and confirmed by UPLC–MS/MS, which indicated that the ic-ELISA established in this work is a reproducible detection assay for nodularin residues in water samples.

## 1. Introduction

**Figure 1:**
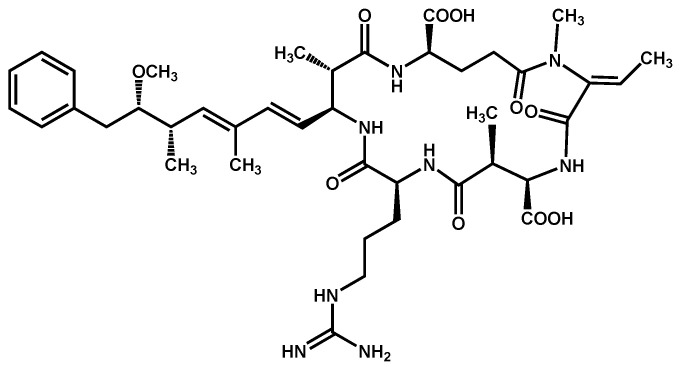
Structure of NOD-R.

Nodularins (NODs) are harmful cyanobacteria toxins produced by *Nodularia spumigena*, especially when blooms brake out in lakes or rivers [1]. So far, different forms of NOD variants have been found. Among these variants, NOD-R is the most abundant and the only one commercially available [2]. NOD-R is a characteristic cyclic peptide composed of five amino acids (-D-MeAsp–L-Y–Adda–D-Glu–Mdhb), in which the Adda structural region ((2S,3S,8S,9S)-3-amino- 9-methoxy-2,6,8-trimethyl-10-phenyl deca-4,6-dienoic acid) is also found in microcystins (MCs), another group of cyanobacteria toxins composed of heptapeptides. Although NOD-R is similar to MCs in structure (Figure 1), it penetrates more easily into hepatocytes than MCs and exhibits stronger acute toxicity [3]. It has been found that NOD-R can inhibit the catalytic subunit of seine/threonine-specific protein phosphatases (PPs) 1 and 2A (PP1 and PP2A) and lead to severe hepatotoxicity [4,5,6]. Meanwhile, NOD-R cannot be completely eliminated via routine water treatment processes because of its water solubility and heat stability; therefore, contaminated drinking water and polluted recreational water might increase the risk of human exposure through intake or skin contact. A guideline value specific for NOD-R in drinking water and recreational water has not yet been established by the World Health Organization (WHO); however, many countries commonly take 1.0 μg/L, the maximum residue limit (MRL) for MC-LR, as a reference [7]. Rapid and sensitive detection methods for NOD-R are required to continuously monitor the level of NOD-R in the environment as well as in drinking water or foods [8].

Recently, several methods for the detection of NOD-R residues have been developed, including high-performance liquid chromatography (HPLC) [9], mass spectrometry (MS) [10], liquid chromatography–mass spectrometry (LC–MS) [11], as well as some rapid detection methods such as immunoassays based on specific antibodies or biosensor assays based on aptamers [12]. Compared with instrumental assay methods, immunoassay technology has the obvious advantages of rapidness, simplicity, and lower cost [13]. For example, only several cents to dollars are commonly spent on each sample when using common ELISA kits, whereas at least tens of dollars are necessary when using UPLC–MS/MS. In addition, antibodies are routinely stable in conformation and common in practical utilization; thus, immunodetection of NOD-R has attracted attention. Both polyclonal antibodies (PAbs) and monoclonal antibodies (MAbs) against NOD-R were obtained, and several detection methods, e.g., indirect competitive enzymatic-linked immunoassay (ic-ELISA), fluorescence polarization immunoassay (FPIA), and optical surface plasmon resonance (SPR) immunosensor detection, were developed, obtaining a limitation of detection (LOD) for NOD-R ranging from 0.051 µg/L to 0.95 µg/L [14,15].

Actually, except for a few examples, most of the antibodies reported above are broad-specificity antibodies originally prepared for MC-LR, but they can recognize also other MCs as well as NOD-R simultaneously. Previously, with MC-LR-KLH as a common immunogen, multi-residue-recognizing PAbs [16] and MAbs [data not shown] were generated in our lab with IC_50_ of 0.29 µg/L and 18 µg/L for NOD-R, respectively.

Obviously, antibodies, as molecular recognition elements, are the core components of immunoassays. In recent years, single-domain antibodies derived from heavy-chain-only antibodies, namely VHH, have been found in camelids, including camels and llamas, as well as VNAR in cartilaginous fishes, including sharks [17]. These antibodies, also called nanobodies (Nbs), have attracted attention for their application in in immunoassays and have shown superiority over traditional PAbs and MAbs because of their small size (-15 kDa) and high solubility and stability [18]. In addition, Nbs can be efficiently generated by the phage display method and can be genetically encoded and expressed in *Escherichia coli* host cells with high yields [19,20]. Until now, several specific Nbs for some chemical contaminants such as parathion [21], aflatoxin B1 [22] and triazophos [23] have been successfully prepared, and related immunoassays have been established.

In this study, an immunized Bactrian camel phage display library was constructed, and then Nbs against NOD-R were selected and prepared. Furthermore, based on optimized reaction conditions, an ic-ELISA for NOD-R in water samples was established. Finally, the performance of the ic-ELISA was measured and validated by UPLC–MS/MS.

## 2. Materials and Methods

### 2.1. Materials and Reagents

Nodularin-R and microcystins (MC-LR, -LA, -LY, -LW, -LF, -YR, -WR, -RR) (Enzo, USA) were used as standards. Nodularin-R-ovalbumin (NOD-R-OVA, prepared in our lab) was the coating antigen. Microcystin-LR-keyhole limpet hemocyanin (MC-LR-KLH, prepared in our lab) was used as the immunogen. An anti-MC-LR monoclonal antibody (anti-MC-LR MAb, prepared in our lab) was used for comparison with the nanobodies. pComb3XSS vector and *E. coli* BL21(DE3) available in our lab were used to construct a recombinant vector and a transformed host strain, respectively. Helper phage M13K07 (New England Biolabs, Ipswich, MA, USA) was used for the construction of a phage displayed nanobody library. The anti-VHH-HRP polyclonal antibody from rabbit (Genscript Bio. Co.Ltd, Nanjing, China) was used as a secondary antibody in ELISA. HisPur Ni-NTA resin (GE Healthcare, Beijing, China) was utilized for protein purification. The primers used in this work were synthesized by Invitrogen Biotechnology Co. (Shanghai, China). Other chemical reagents were provided by Sigma (St. Louis, MS, USA) and Thermo Fisher Scientific (Thermo, Waltham, MA, USA).

### 2.2. Instruments

A NanoDrop 2000C spectrophotometer (Thermo, Waltham, MA, USA) was used to measure absorbance in the ultraviolet–visible spectrum. Centrifugation of phages and bacteria was performed using SORVALL LYNX 4000 centrifuges (Thermo, Waltham, MA, USA). Biologic LP (Bio-Rad, Hercules, CA, USA) was used to purify VHH antibodies. The absorbance values in ELISA were measured using a Multiskan MK3 microplate reader (Thermo Scientific, Waltham, MA, USA).

### 2.3. Construction of a Phage-Displayed Nanobody Library

The procedure for constructing the nanobody library by the phage display technology was similar to that previously published [24]. In practice, a three-year-old Bactrian camel was immunized with 500 μL of MC-LR-KLH(1.0 mg/mL) emulsified with Freund’s adjuvant at a ratio of 1:1 (*v/v*) every two weeks for a total of 6 times. Particularly, the first time of immunization was performed with complete adjuvant, and the boost immunizations with incomplete adjuvant. One week after the sixth immunization, 100 mL of fresh peripheral blood was collected, and lymphocytes were isolated, followed by RNA extraction. After the synthesis of cDNA by RT-PCR, the VHH genes were amplified by two-step nested PCR using the primers shown in Table 1. The VH−CH1−CH2 and VHH−CH2 regions were firstly amplified using the primers CALL001and CALL002. Next, the primers Fr4-*Sfi*I and Fr1-*Sfi*I were used to amplify the VHH genes. The VHH genes and the pComb3XSS vector were digested using the restriction endonuclease *Sfi*I, ligated, and transfected into competent *E. coli* ER2738 cells. All transformants were collected by scraping from LB–agar plates containing ampicillin and tetracycline. After infection with helper phage M13K07, a phage-displayed Nb library was constructed. The capacity and diversity of the constructed library were identified by sequence analysis of 20 randomly selected clones.

### 2.4. Biopanning and Identification of Nanobody Clones for NOD-R

Four rounds of biopanning in 96-well microtiter plates were performed, as described in references to select Nb clones for NOD-R [25]. In practice, the antigen NOD-R-OVA was coated at decreasing concentrations (10, 2.5, 0.5, 0.1 μg/mL) in four rounds of biopanning at 4 ℃ with overnight incubation. Meanwhile, 1 mg/mL of OVA and BSA was coated in the parallel wells under the same condition (4 ℃, overnight). After shaking out the excess coating solution, a 1% gelatin solution was used to block the coated wells at 37 ℃ for 2 h. In each round of biopanning, 100 µL of phage library (about 1 × 1011 pfu/mL) was introduced into OVA-wells and BSA-wells in turn, for 1 h at 37 ℃, to pre-absorb the non-specific phages; then, the supernatant was transferred to NOD-R-OVA-coated wells and incubated for another 1 h at 37 ℃. After washing with PBST for six times, elution was conducted by incubating with decreasing concentrations of NOD-R (2, 0.5, 0.1 μg/mL) and 10 mg/mL of trypsin under gentle shaking at 37 ℃ for 1 h in each round. Finally, 10 µL of elution solution was used to test the phage titer, and the remainder was amplified for the next round of biopanning.

After four rounds of biopanning, 20 clones were picked out randomly and induced by 1.0 mmol/L isopropylthio-β-D-galactoside (IPTG) to express Nbs in 96-deep-well plates at 37 ℃ with shaking at 250 rpm for 16 h. Then, ic-ELISA was used to identify the positive clones. Furthermore, the positive clones were subjected to DNA sequencing.

### 2.5. Expression and Characteristics of Nanobodies

The recombinant vector pComb3XSS-vhh with the NOD-R VHH gene was extracted from the positive *E. coli* ER2738 clones and then transfected into competent cells of the host strain *E. coli* BL21(DE3) to express specific Nbs. For scaled-up expression, 10 mL of the overnight culture was added to 1 L of LB medium (with 100 mg/mL ampicillin) and incubated at 37 °C with shaking at 250 rpm. When the absorbance of the culture at a wavelength of 600 nm (A_600nm_) reached 0.6–0.8, 1 mM IPTG was added to induce the expression of the target protein, followed by continuous incubation overnight. Then, the bacteria precipitate was harvested after centrifugation at 12,000 rpm for 15 min. The extraction solution (0.2 M Tris-HCl, pH 8.0, 0.5 mM EDTA, 0.5 M sucrose) was added, and the soluble proteins were isolated via cycles of freezing and thawing and by using the osmotic pressure method. The expressed Nbs were purified using the HisPur Ni-NTA resin, followed by 15% SDS-PAGE and Western blotting identification.

To assess the thermostability of the candidate Nbs, 1 mg/mL of purified Nbs was separately incubated at different temperatures, i.e., 25, 37, 50, 65, 80, 90 °C for 10 min, or at 90 °C for 10, 20, 30, 40, 50, and 60 min. The organic solvent tolerance of Nbs was evaluated using different concentrations (10%, 20%, 30%, 40%, 50%, 60%, 70%, 80%, and 100%) of methanol (MeOH) or acetonitrile. PBS buffer (10 mm PBS, pH = 7.0) was used as the dilution reagent (*v/v*), and Mc-MAb was used as the control.

### 2.6. Establishment of an Indirect Competitive ELISA Based on the Nanobody N56

The ic-ELISA for NOD-R was developed under optimized reaction conditions. The concentrations of N56 and coating antigen NOD-R-OVA were optimized by checkboard titration. Different buffers (PB, PBS, PBST, Tris-HCl, HEPES), pH values (6.5, 7.0, 7.5, 8.0), and ionic strengths of PBS (5 mM, 10 mM, 20 mM, 40 mM) were chosen to optimize the conditions based on absorbance values of approximately 1.0 in ELISA at 450 nm wavelength or on the Amax (maximal absorbance)/IC_50_ ratio. Then, the established ic-ELISA was performed as follows. In practice, 100 µL/well of NOD-R-OVA solution was used to coat a 96-well plate, which was incubated overnight at 4 ℃ and then blocked with 3% BSA in PBS buffer for 1 h. After five washings with PBST buffer, 50 μL/well of an N56 solution at optimal concentration and 50 μL/well of NOD-R at different concentrations were added into the microwells, which were incubated for 40 min at 37 ℃ with a slight oscillation. After additional five washings, 100 µL/well of rabbit anti-VHH-HRP polyclonal antibody (1:5000 dilution) was added in the microwells, and incubation was carried out at 37 ℃ for 30 min. After 5 more washings, 100 µL/well of TMB peroxidase substrate buffer was added, and the enzyme reaction was stopped after a 10 min incubation by the addition of 50 µL/well of 10% H_2_SO_4_ (*v/v*). The absorbance values of the microplates were measured using a microplate reader at 450 nm wavelength. Furthermore, a standard curve was obtained based on the established four-parameter logistic equation utilizing Origin 8.5 (Origin Lab Corp., Northampton, MA, USA). Consequently, the limit of detection (LOD) was defined as the concentration with 10% inhibitory activity and the half-maximal inhibitory concentration (IC_50_) as the concentration with 50% inhibitory activity. The rate of cross reactivity was calculated according to a previously formula, namely, CR (%) = IC_50_ (NOD-R, µg/L)/IC_50_ (other toxins, µg/L) × 100.

### 2.7. Analysis of Water Samples by Ic-ELISA for NOD-R Detection 

Tap water was from Guangdong Provincial Key Laboratory of Food Quality and safety, College of Food Science, South China Agricultural University (Guangzhou, China), lake water was from NingYin Lake in South China Agricultural University, and river water was from CheBei river. After filtering through a 0.22 μm nitrocellulose membrane, all water samples (5 mL) were spiked with different concentrations of NOD-R standard obtaining concentrations of 0, 0.5, 1, 2 µg/L for assessment of the recovery. Furthermore, the spiked samples were analyzed by the established ic-ELISA. According to the ic-ELISA standard curve, the values of mean recovery, SD, and CV were calculated.

The N56-based ic-ELISA was verified by UPLC–MS/MS operated at the Guangzhou Institute for Food Control, China. The conditions were established according to the literatures [16]: Accucore aQ C18 column (150 mm × 2.1 mm, 2.6 μm, Thermo Scientific, USA) was used for chromatographic separation. The mobile phase A was a MeOH solution containing 0.1% formic acid (*v/v*) and 5 mmol/L ammonium formate, while the mobile phase B was an aqueous solution with the same constituents as the mobile phase A. The gradient elution was 0–0.5 min, 80% B; 0.5–4 min, 80–25% B; 4–7 min, 25–0% B; 7–7.5 min, 0% B; 7.5–8 min, 0–80% B; 8–10 min, 80%B. The flow rate was 0.3 mL/min, and the injection volume of each sample was 5 µL. The temperature of the column oven was set at 40 °C. The mass spectra were obtained with the AB TRIPLE QUAD 4500 mass spectrometer under positive ionization mode, with the following parameters’ values: curtain gas flow rate, 30 L/min; nebulizer gas flow rate, 50 L/min; auxiliary gas flow rate, 55 L/min; collision gas, 8 L/min; spray voltage, 5.5 kV.

## 3. Results and Discussion

### 3.1. Selection of Anti-NOD-R Nanobodies from the Phage Display Library

**Figure 2:**
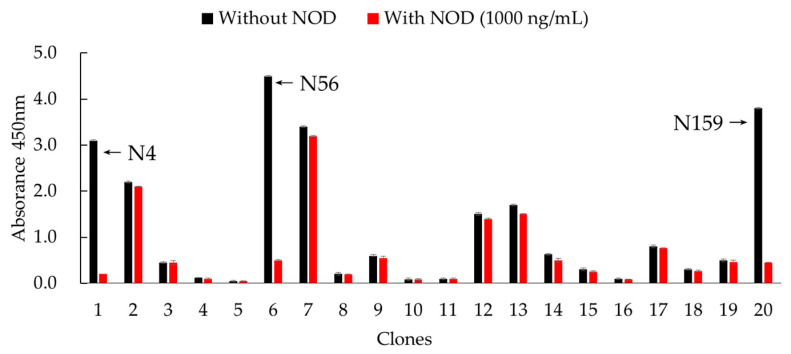
Positive clones identified by ic-ELISA.

**Figure 3:**
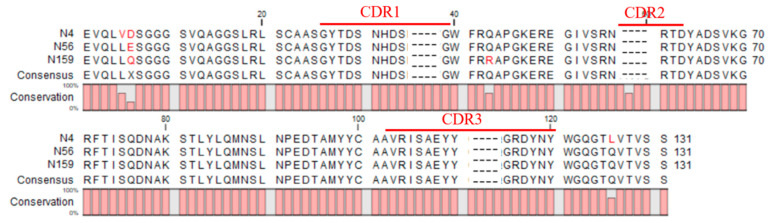
Sequence alignments of the three positive clones.

Considering that MC-LR-KLH previously prepared in our lab as a common immunogen can induce excellent immunization and has been successfully used to generate specific PAbs and MAbs which could recognize NOD-R, in this work, this molecule was chosen as the immunogen for the camel [16]. After six inoculations, the serum titer was up to 1:64,000, and inhibition of NOD-R was higher than 80%. By two-step nested PCR, VHH genes were amplified, and a bacterial library with the capacity of 8.0 × 10^7^ cfu/mL was constructed. Then, rescued by helper phage M13K07, the titer of phage-displayed Nbs library was about 1.0 × 10^12^ pfu/mL. Furthermore, after four rounds of biopanning, three clones, namely, N4, N56, and N159, were identified as positive ones and exhibited recognition activity for NOD-R (Figure 2). The inhibition rates of these three clones identified by ic-ELISA were all higher than 80%. By sequence alignment analysis, these three clones, composed of 131 amino acid residues, were confirmed to possess the characteristics of Nbs and to have high similarity, except for a few residues in frame regions (FRs) (Figure 3). Therefore, they were considered as the same category of Nbs, and N56 was chosen for further analysis.

### 3.2. Preparation and Characterization Analysis of the Nanobody N56

**Figure 4:**
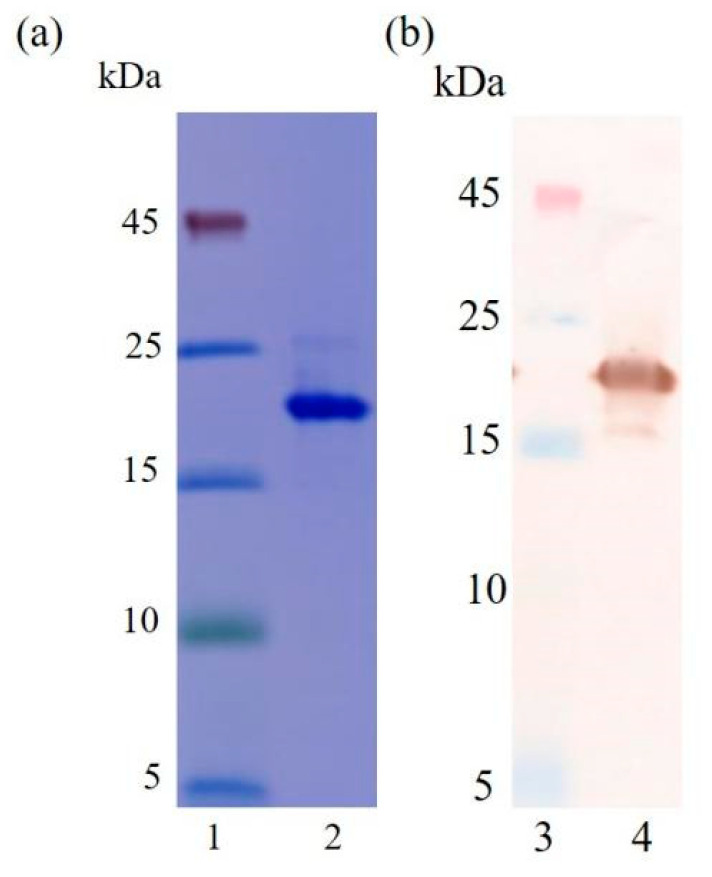
Identification of N56 by SDS-PAGE and Western blotting. (**a**) SDS-PAGE: lane 1, marker; lane 2, purified N56. (**b**) Western blotting: lane 3, marker; lane 4, purified N56.

To better investigate the characteristics of the nanobody N56, it was expressed in 1L of LB medium and purified using the HisPur Ni-NTA resin. The purified N56 were identified by 15% SDS-PAGE (Figure 4a) and Western blotting (Figure 4b). The results showed that the molecular weight of N56 is around 17 kDa, with purity of more than 90%, and the yield was about 4.5 mg/L. It has been observed that Nbs can be highly expressed in *E. coli*, yielding from more than 10 mg to tens of milligrams, thanks to their low molecular weight and good solubility [26]. However, in our work, N56 showed a relatively low yield, not higher than 5 mg/L. We thought it might be related to the ratio of hydrophobic amino acids (22%) in its composition, but after comparison with other Nbs, e.g., Nb-T3-15 (for tetrabromobisphenol A) [27] or Nb-3F9 (for tenuazonic acid) [28], which gave yields up to 30 mg/L and just less then 1 mg/L under the same expression conditions, respectively (Figure S1), this hypothesis was discarded because Nb-T3-15 has a higher hydrophobic amino acids ratio than Nb-3F9. Therefore, maybe the location of the hydrophobic amino acids in the 3D structure of Nbs plays a more important influence on the solubility of nanobodies then their expression level in *E. coli* host cells.

**Figure 5:**
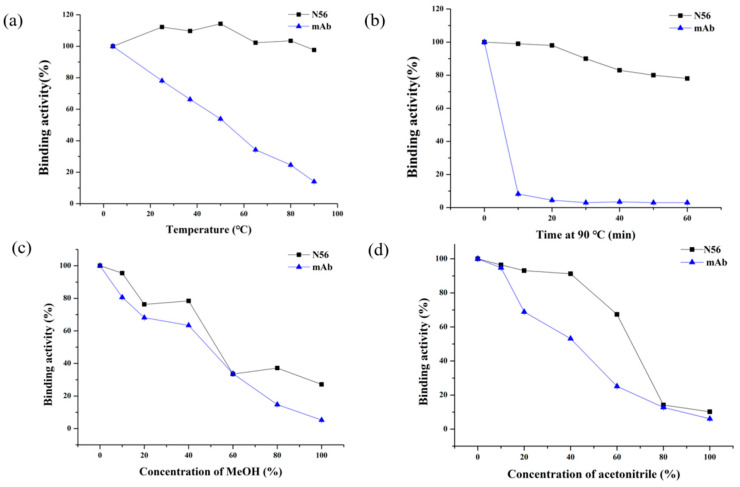
Thermostability and organic solvent tolerance of N56 and Mc-MAb by ic-ELISA: (**a**) N56 and Mc-MAb (1 mg/mL) were incubated at 25, 37, 50, 65, 80, and 90 ℃ for 10 min; (**b**) N56 and Mc-MAb (1 mg/mL) were incubated at 90 ℃ for 10, 20, 30, 40, 50, and 60 min; a series concentrations (10%, 20%, 30%, 40%, 50%, 60%, 70% and 80%) of (**c**) methanol (MeOH) and (**d**) acetonitrile were used to dilute (*v/v*) N56 and Mc-Mab (*n* = 3).

Furthermore, the thermostability and organic solvent tolerance of N56 was evaluated with Mc-MAb as a control. After incubation at different temperatures, from 4 ℃ to 90 ℃, for 10 min, N56 could maintain a binding activity higher than 90%, whereas Mc-MAb lost its activity very quickly. Especially at 25 ℃ (RT) or 37 ℃, two commonly used temperatures during practical detection, N56 exhibited full activity, while Mc-MAb lost nearly 30% of its activity (Figure 5a). Even more, N56 was stable at the high temperature of 90 ℃ for 1 h, retaining 80% of its activity (Figure 5b). In addition, the tolerance of N56 for the organic solvents methanol (MeOH) and acetonitrile was determined. As shown in Figure 5c,d, with increasing concentration of MeOH or acetonitrile, both N56 and Mc-MAb began to lose their activities; however, N56 as a whole had superior performance than Mc-Mab, in particular, it showed better tolerance to acetonitrile. Generally, the disulfide bonds formed by cysteine residues present in Nbs are thought to contribute to its excellent stability. According to the sequence of N56, four cysteine residues (cys22, cys37, cys100, cys103) might form two disulfide bonds and maintain the stability of N56 structure in harsh environments, at either high temperature or in the presence of organic solvents (Figure 3). This phenomenon was also observed for other reported Nbs for low-molecular-weight chemical contaminants [29].

### 3.3. Establishment of an Ic-ELISA Based on the Nanobody N56

**Figure 6:**
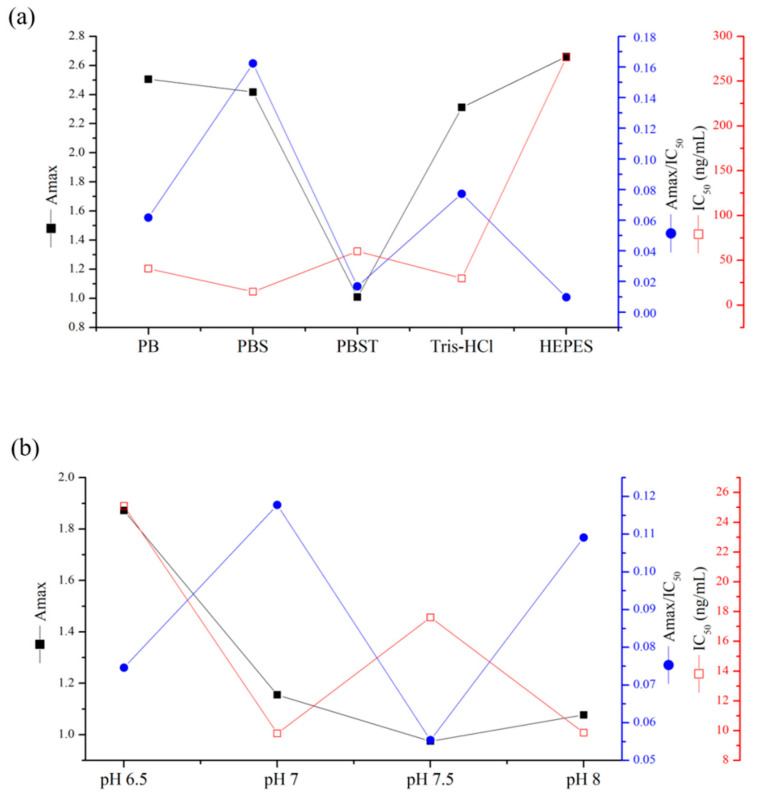

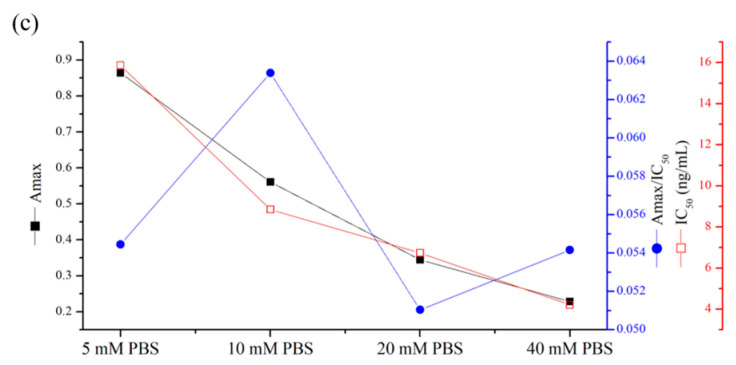
Optimization of assay conditions for the establishment of ic-ELISA: (**a**) types of buffers (PB, PBS, PBST, Tris-HCl, and HEPES), (**b**) pH value (6.5, 7.0, 7.5, and 8.0), and (**c**) ionic strength (5 mM, 10 mM, 20 mM, and 40 mM PBS). The conditions were evaluated by the Amax (maximal absorbance)/IC_50_ ratio.

**Figure 7:**
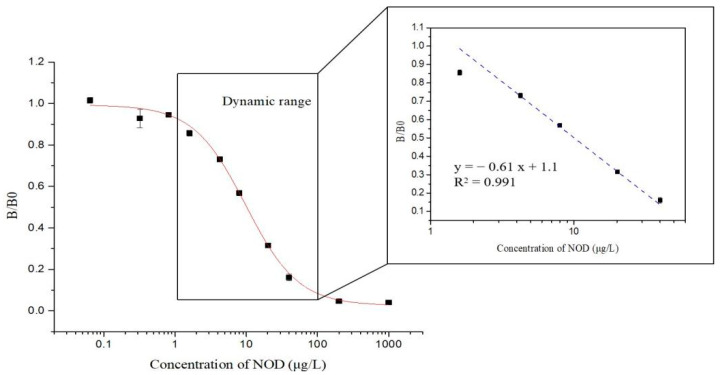
Standard curves of ic-ELISA for NOD-R based on purified N56 under the optimized reaction conditions. The standard curves were normalized by expressing the experimental absorbance values (B) as B/B_0_, where B_0_ is the absorbance value at zero analyte concentration (*n* = 3).

In order to develop an ic-ELISA for NOD-R with N56 as the recognition element, various reaction conditions, such as concentrations of nanobody and coating antigen, buffers, pH, and ionic strength, were optimized (Figure 6a–c). After the checkboard titration test, the optimal concentrations of N56 and NOD-R-OVA were determined to be 0.125 µg/mL and 2 µg/mL, respectively. The other conditions were further optimized with the highest Amax/IC_50_ as the selection criterion [28]. Consequently, 10 mM PBS buffer at pH 7.0, at a relative lower ionic strength under a neutral reactive environment, was confirmed as the most suitable buffer for the establishment of ic-ELISA. Finally, an ic-ELISA standard curve based on N56 for NOD-R was established (Figure 7). The IC_50_ value was 9.94 µg/L, and the linear range of detection was 1.74–56.66 µg/L with a limit of detection (LOD) of 0.67 µg/L, which was lower than that of Mc-MAb for NOD-R (1.25 µg/L). This LOD value can also satisfy the detection requirement of 1 µg/L in drinking water.

Besides, the N56-based assay showed relatively significant cross reactivity for MCs. This might result from the common special Adda structural regions ((2S,3S,8S,9S)-3-amino- 9-methoxy-2,6,8-trimethyl-10-phenyl deca-4,6-dienoic acid). In this work, NOD-R-OVA was used as the coating antigen, and NOD-R at decreasing concentration as the competitive drug to increase the specificity of the eluted phages; yet, the generation of VHHs against the Adda structure in camel by the immunogen MC-LR-KLH was possible, and these antibodies would consequently recognize NOD-R as well as MCs. In addition, among the MCs capable of binding N56, MC-LR, -YR, -RR, -WR exhibited higher cross reactivity than other MCs (Table 2). This is because these four MCs have an arginine residue as NOD-R besides the Adda region. From another perspective, high cross reactivity might be helpful in the detection of multiple residues of cyanobacteria toxins. It was reported that VHHs for MC-LR prepared by Pírez-Schirmer et al. [30] or Xu et al. [31] could recognize other MCs, but there are no data about their cross reactivity with NOD-R. In this study, we, for the first time, obtained N56 that is able to detect NOD-R and MCs, i.e. two groups of cyanobacteria toxins.

### 3.4. Water Sample Analysis by ELISA and UPLC–MS/MS

The N56-based ic-ELISA was further employed to analyze water samples spiked with NOD-R at three concentrations (0.5, 1, 2 µg/L). As shown in Table 3, the average recovery ranged from 111.0% to 119.5%, with CVs of 2.0–5.8%. The value of the correlation coefficient (R^2^) between ic-ELISA and UPLC–MS/MS was up to 0.999, indicating that the established N56-based ic-ELISA is accurate and reliable for detecting NOD-R in water samples.

## 4. Conclusions

In this work, three Nbs for NOD-R were selected from a Bactrian camel phage display library, and N56 showed high stability and good affinity. Then, an ic-ELISA method for NOD-R detection was established, with a LOD lower than 1.0 μg/L, the generally reference value for cyanobacteria toxins. In a thorough comparison with UPLC–MS/MS, the ic-ELISA method exhibited good accuracy and reliability, which suggests its potential for the development of a rapid detection kit for the safety detection of toxins based on N56.

## Figures and Tables

**Table 1:** Primers’ sequences used to amplify the VHH genes (W = A or T, S = G or C, M = A or C, R = G or A).

Primer Names	Nucleotide Sequences (5′→3′)
CALL001	GTCCTGGCTGCTCTTCTACAAGG
CALL002	GGTACGTGCTGTTGAACTGTTCC
Fr4-*Sfi*I	ACTGGCCCAGGCGGCCGAGGTGCAGCTGSWGSAKTCKG
Fr1-*Sfi*I	ACTGGCCGGCCTGGCCTGAGGAGACGGTGACCWGGGTC

**Table 2:** CRs of N56 for MCs analogues (*n* = 3).

Algal Toxins	Structure	IC_50_(µg/L)	CRs
NOD	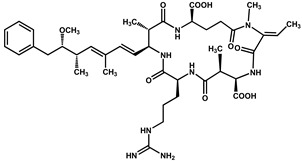	9.94	100.0%
MC-LR	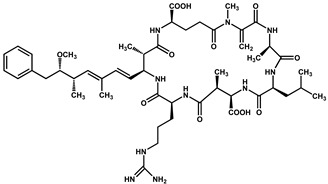	8.24	120.6%
MC-LA	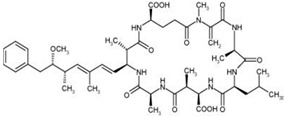	42.41	23.4%
MC-LY	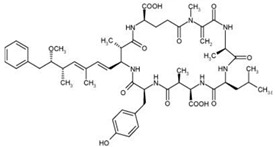	60.78	16.4%
MC-LW	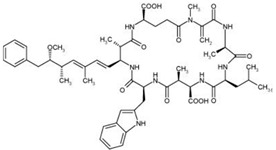	180.81	5.5%
MC-LF	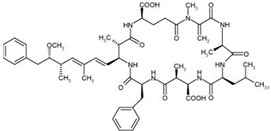	113.79	8.7%
MC-YR	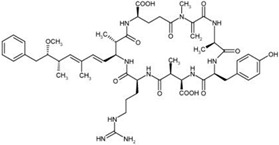	7.85	126.6%
MC-WR	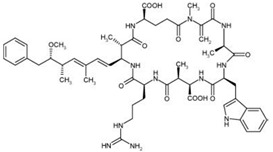	13.50	73.6%
MC-RR	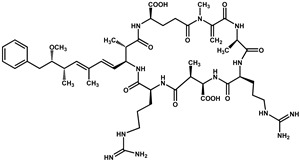	11.87	83.7%

**Table 3:** Reproducibility and accuracy of NOD-R from spiked water samples by ic-ELISA and LC–MS/MS (*n* = 3).

	Ic-ELISA			UPLC-MS/MS			R^2^
Spiked Level (µg/L)	Found ± SD (µg/L)	Recovery (%)	CV (%)	Found ± SD (µg/L)	Recovery (%)	CV (%)	
0	/	/	/	/	/	/	0.999
0.5	0.59 ± 0.02	117.7	3.6	0.53 ± 0.01	106.0	1.9	
1	1.11 ± 0.07	111.0	5.8	1.07 ± 0.02	107.0	1.9	
2	2.39 ± 0.02	119.5	2.0	2.33 ± 0.04	116.5	1.5	

## Supplementary Materials

The following are available online at https://www.mdpi.com/article/10.3390/foods10112758/s1, Figure S1: Comparison of the expression levels of three nanobodies (Nb-T3-15, Nb-56, Nb-3F9) by SDS-PAGE; Figure S2: Results of ethical review of animal experiments. (**a**) Original copy. (**b**) English translation; Table S1: Abbreviation list.
