# Supplementary material for: Preparation of Specific Nanobodies and Their Application in the Rapid Detection of Nodularin-R in Water Samples
Source: Foods. 2021 Nov 10;10(11):2758. doi: 10.3390/foods10112758 (PMC8622565; doi:10.3390/foods10112758)
Supplement: Supplementary file 1 [file foods-10-02758-s001.zip › foods-1418850- supplementary.pdf]

Supplementary

Preparation of Specific Nanobodies and Their Application in the Rapid Detection of Nodularin-R in Water Samples

Jinyi Yang [1,†], Rui Si [1,†], Guangpei Wu [1], Yu Wang [2], Ruyu Fang [1], Fei Liu [1], Feng Wang [1], Hongtao Lei [1], Yudong Shen [1], Qi Zhang [3] and Hong Wang [1,*]

[1] Guangdong Provincial Key Laboratory of Food Quality and Safety, National-Local Joint Engineering Research Center for Processing and Safety Control of Livestock and Poultry Products, College of Food Science, South China Agricultural University, Guangzhou 510642, China; yjy361@163.com (J.Y.); huanongxiaosi@163.com (R.S.); wgphavoc@163.com (G.W.); 18819259799@163.com (R.F.); m13476079515@163.com (F.L.); wangfeng_sp@163.com (F.W.); hongtao@scau.edu.cn (H.L.); shenyudong@scau.edu.cn (Y.S.)

[2] Guangzhou Institute of Food Inspection, Guangzhou 510080, China; xxwangyu@163.com

[3] Oil Crops Research Institute, Chinese Academy of Agricultural Sciences, Xudong 2nd Road No.2, Wuchang District, Wuhan 430062, China; zhangqi01@caas.cn

* Correspondence: gzwhongd@163.com; Tel.: +86-20-85283448; Fax: +86-20-85280270

† These authors contribute equally to this work.

Citation: Yang, J.; Si, R.; Wu, G.; Wang, Y.; Fang, R.; Liu, F.; Wang, F.; Lei, H.; Shen, Y.; Zhang, Q.; et al. Preparation of Specific Nanobodies and Their Application in the Rapid Detection of Nodularin-R in Water Samples. *Foods* **2021**, *10*, 2758. https://doi.org/foods10112758

Academic Editor: Juliana Moura Luna

Received: 28 September 2021
Accepted: 5 November 2021
Published: 10 November 2021

Publisher's Note: MDPI stays neutral with regard to jurisdictional claims in published maps and institutional affiliations.

Copyright: © 2021 by the authors. Submitted for possible open access publication under the terms and conditions of the Creative Commons Attribution (CC BY) license (https://creativecommons.org/licenses/by/4.0/).

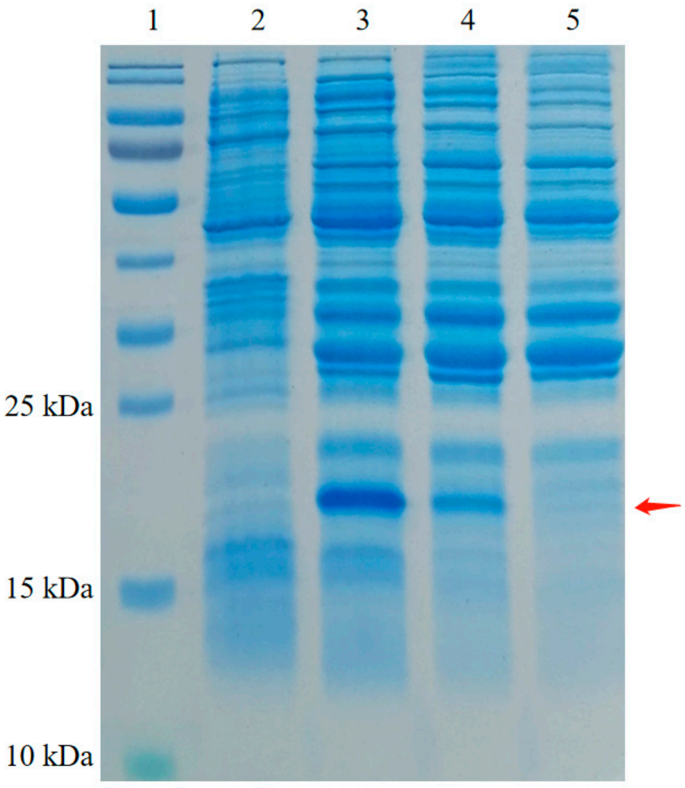

Figure S1. Comparison of expression levels of three nanobody (Nb-T3-15, Nb-56, Nb-3F9) by SDS-PAGE. lane 1, marker; lane 2, wild type *E.coli* BL21(DE3); lane 3, Nb-T3-15; lane 4, Nb-56; line 5, Nb-3F9.

Table S1. Abbreviation list.

Materials	abbreviation
Nodularins	NODs (NOD-R)
Microcystins	MCs (MC-LR, -LA, -LY, -LW, -LF, -YR, -WR, -RR)
Nodularin-R-ovalbumin	NOD-R-OVA
Microcystin-LR-keyhole limpet hemocyanin	MC-LR-KLH
Anti-MC-LR monoclonal antibody	Anti-MC-LR MAb
indirect competitive enzyme-linked immuno-sorbent assay	ic-ELISA
protein phosphatases (PPs) 1 and 2A	PP1 and PP2A
maximum residue limit	MRL
high performance liquid chromatography	HPLC
mass spectrometry	MS
polyclonal antibodies	PAbs
monoclonal antibodies	MAbs
fluorescence polarization immunoassay	FPIA
isopropylthio-β-D-galactoside	IPTG
limit of detection	LOD
maximal inhibitory concentration	IC_{50}
cross reactivity	CR

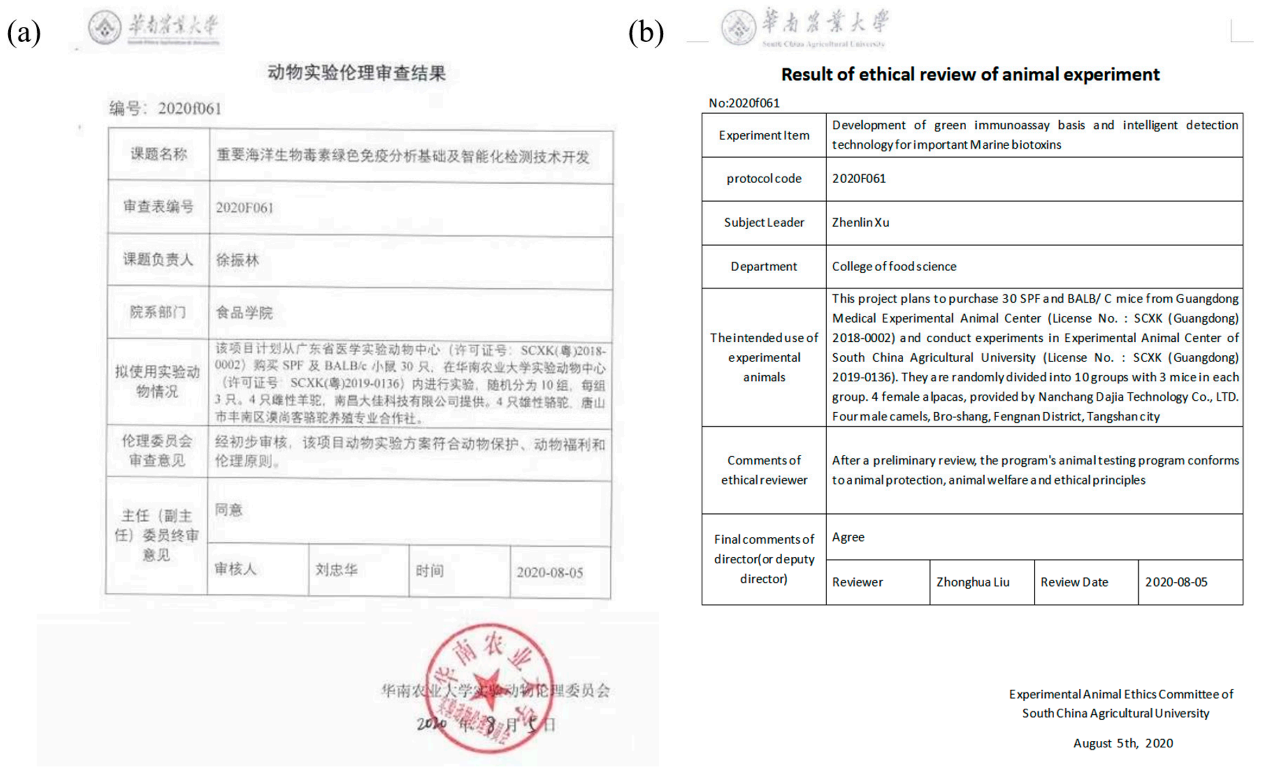

(a)

动物实验伦理审查结果

编号：2020f061

课题名称	重要海洋生物毒素绿色免疫分析基础及智能化检测技术开发
审查表编号	2020F061
课题负责人	徐振林
院系部门	食品学院
拟使用实验动物情况	该项目计划从广东省医学实验动物中心（许可证号：SCXK(粤)2018-0002）购买 SPF 及 BALB/c 小鼠 30 只，在华南农业大学实验动物中心（许可证号：SCXK(粤)2019-0136）内进行实验，随机分为 10 组，每组 3 只。4 只雌性羊驼，南昌大佳科技有限公司提供。4 只雄性骆驼，唐山市丰南区溴尚客骆驼养殖专业合作社。
伦理委员会审查意见	经初步审核，该项目动物实验方案符合动物保护、动物福利和伦理原则。
主任（副主任）委员终审意见	同意<br>审核人 刘忠华 时间 2020-08-05

华南农业大学实验动物伦理委员会

2020 年 8 月 5 日

(b)

Result of ethical review of animal experiment

No:2020f061

Experiment Item	Development of green immunoassay basis and intelligent detection technology for important Marine biotoxins
protocol code	2020F061
Subject Leader	Zhenlin Xu
Department	College of food science
The intended use of experimental animals	This project plans to purchase 30 SPF and BALB/ C mice from Guangdong Medical Experimental Animal Center (License No. : SCXK (Guangdong) 2018-0002) and conduct experiments in Experimental Animal Center of South China Agricultural University (License No. : SCXK (Guangdong) 2019-0136). They are randomly divided into 10 groups with 3 mice in each group. 4 female alpacas, provided by Nanchang Dajia Technology Co., LTD. Four male camels, Bro-shang, Fengnan District, Tangshan city
Comments of ethical reviewer	After a preliminary review, the program's animal testing program conforms to animal protection, animal welfare and ethical principles
Final comments of director(or deputy director)	Agree<br>Reviewer Zhonghua Liu Review Date 2020-08-05

Experimental Animal Ethics Committee of South China Agricultural University

August 5th, 2020

Figure S2. Results of ethical review of animal experiments. (**a**) Original copy. (**b**) English translation.
